# Supplementary material for: Developing engaged and ‘teamful’ leaders: A randomized controlled trial of the 5R identity leadership program
Source: PLoS One. 2023 May 25;18(5):e0286263. doi: 10.1371/journal.pone.0286263 (PMC10212178; doi:10.1371/journal.pone.0286263)
Supplement: Attachment — Submitted filename: Response to Recviewers PLoSONE 5R v1.2.docx [file pone.0286263.s001.docx]

**Key Editorial points**

E•1. *Developing a more theoretical rationale for the study and one which provides a stronger narrative.*

The key points that you and the reviewers raised related to the need to tighten the theoretical rationale for the study. This was something we were happy to do, having previously cut some discussion of the theoretical rational in order ensure the paper was not overly long. Specifically, we have elaborated on the rationale for the study in a number of places in the Introduction. In particular, on p.6 we now note that:

*This approach differs from prevailing approaches to leader development which focus largely on improving the skills and mindsets of individual leaders. Work which has evaluated such programs indicates that they generally succeed in developing a person’s leader identity (their sense of themselves as a leader). In contrast to this focus on ‘me’ and ‘I’, the social identity approach embraces the idea that leadership is fundamentally a group process requiring leaders to look outwards towards their teams and to develop sense of ‘we’ and ‘us’.*

E•2. *Additional files*

In your letter you also asked us to include (a) A marked-up copy of your manuscript that highlights changes made to the original version (labeled 'Revised Manuscript with Track Changes') and (b) An unmarked version of your revised paper without tracked changes. You said we should upload this as a separate file labeled 'Manuscript'. We have done both these things.

E•3. *Style requirements*

We have followed your instructions and ensured that the manuscript follows the journal’s style requirements — especially by reformatting references and using US spelling throughout.

E•4. *Conflict of interest*

We confirm that our stated conflict of interest does not alter our adherence to all *PLoS ONE* policies on sharing data and materials.

E•5. *Details of data repository*

We have provided an anonymized link in the paper to the data which has now been loaded onto the platform of the Open Science Foundation. This is available here: https://osf.io/e82bd/?view_only=2d846d686e844a5ebd28347fbed32396

E•6. *Removal of reference*

We have removed reference to the unpublished manuscript by Gopinathan (2017).

**Specific points raised by reviewers**

**Reviewer 1**

*General comment.* We very much appreciate the trouble that this reviewer had gone to in reviewing the paper. Clearly an expert in the field, their observations seemed appropriate and well judged. We appreciate the general statement that “The question addressed by the paper is important theoretically and practically, with the potential to contribute to different literature streams” and the reviewer’s sense that the paper is well-written and has something interesting to say, but agree that the paper could be sharpened by reflection on the range of points they make. These were as follows:

R1•1 *Clearer discussion of the theoretical framework that informs the paper*

The reviewer asked us to “locate [our] work in a broader context” of work on leadership and leadership development programs in a way that makes it clear what the distinctive theoretical of the paper (and the 5R program) is.

The reviewer’s points here are very well taken, and in revising the manuscript we have tried to engage closely with this issue, in particular by spelling out the differences between our ‘we-focused’ approach and the ‘I-focus’ on programs that focus on developing leader identity.

R1•2a *The issue of common method variance (CMV)*

The reviewer notes that “although the data was collected at different points in time and there is an intervention, it is still the same source and the same criteria, which is a significant concern for common method variance”. It is certainly the case that the study’s reliance on self-report data was a limitation (as we note in the Discussion). As we note in the text though, (on p.9) this was an issue we attempted to address by including a measure of participants’ “leader identity pursuit” as a control variable that has superficial similarity to our target dependent variable (identity leadership knowledge), but which taps different theoretical processes (as discussed by Haslam et al., 2022). In line with the logic of non-equivalent dependent variable design (Cook et al., 1979; see also Frese et al., 2003), this allows us to rule out the possibility that any changes we observe arise from non-specific responses to the intervention such as CMV (Podsakoff et al., 2012). In revising the paper in both the Introduction (p.9) and the Discussion (p.24) we have now made an explicit reference to the fact that this strategy was designed to address this problem.

R1•2b *Construct measures*

The reviewer makes the point that a lot of our measures related to leaders’ perceptions of the team (e.g., team psychological safety, team engagement). We agree that this is somewhat unorthodox; however, in line with the goal of 5R — which as we note in the Introduction was to encourage leaders to focus out on their team and to engage with it — we think that these measures make sense in so far as they capture the impact of the leader (and their participation in 5R) on team function. Indeed, in line with the idea that participation in 5R would make leaders mindful of their team (i.e., ‘teamful’), we think that it is important to capture the extent to which the program was perceived by leaders to have these effects — which the results suggest that it did.

R1•3 *Inclusion of effects sizes*

The reviewer points out that because we had a relatively small sample size but the effects were still statistically significant, this suggests that the effect sizes were quite large, and that it would be good to report them in the paper. This was a very good observation, and, as the reviewer recommends, we now report effect sizes for all effects. As the reviewer anticipated, most of the effects in our treatment conditions were of moderate-to-large size, whereas those in the control condition tended to be small (and sometimes in the opposite direction).

R1•4 *Extended Discussion*

Related to R1•1, the reviewer observes that the Discussion was a bit underdeveloped and could do with being fleshed out to address the significance of the paper for the broader leadership literature. Again, this makes a lot of sense, and we have attempted to develop the Discussion along the lines they suggest. In particular, we have added this text at the start of the Discussion as well as a table (Table 3) that summarizes some of the key differences between 5R and traditional approaches to leadership:

*Where traditional approaches to leader(ship) training and development focus on leaders in isolation and often in contexts removed from their normal sphere of activity in ways that aim to build leader identity, 5R encourages leaders to engage directly with the groups they are attempting to lead and gives them the skills to do so with confidence in ways that build identity leadership. In this way, and in line with suggestions that leadership development should focus on the specific contexts in which leaders operate, the program is designed to help leaders mobilize followers (the team members for whom leaders have responsibility) rather than to exclude them from the leadership process and the broader dynamics of organizational development and change.*

**Reviewer 2**

*General comment.* This reviewer is also clearly very knowledgeable about social identity and the field of leadership and raises a range of important points in the process of providing very valuable feedback. We appreciate their general judgment that “I very much liked reading this paper and think it presents a very nice study on an important topic, namely a randomized control trial of an identity leadership intervention.” as well as their statement that “The manuscript is well written and the hypotheses are well developed”. Like Reviewer 1, though, s/he makes a series of good points that needed to be addressed in the revision. These were as follows:

R2•1. *Downplay independence of instructors*

The reviewer that we suggest downplay the independence of the trainers/facilitators as the graduate students performing these roles were themselves trained by the authors. This is a fair point, and we have followed the reviewer’s suggestion and downplayed this in both the abstract and the manuscript as a whole.

R2•2. *Provide more detail about the study in the abstract*

The reviewer asked us to provide a bit more detail about the study’s design in the abstract. As they suggested, we have included the *N*s for each condition and also provided more details about the study’s findings.

R2•3. *Clarification of results on leader identity pursuit measure*

The reviewer asks us to clarify our discussion of the results on the control measure (leader identity pursuit) by explaining how the findings differed across the two treatment conditions. As they suggest, we have done this on p.26 by noting that insofar as we expected participation in 5R to reduce participants’ leader identity pursuit, the fact that this was seen in the Facilitated (but not the Self-Directed) condition suggests that some of the lessons of the program were internalised more by participants in the facilitated version of the program than by those in the self-directed version.

R2•4. *Note that the absence of follower data is a limitation*

As the reviewer (and R1) notes, we agree that ideally we would have gathered data from followers to assess the impact of leaders’ participation in 5R on them and the group as a whole. This had certainly been our intention, but it proved too logistically challenging. However, as we now note in the revised manuscript (on p.28), it is our intention to do this in future trials.
